# Zanubrutinib monotherapy in relapsed/refractory mantle cell lymphoma: a pooled analysis of two clinical trials

**DOI:** 10.1186/s13045-021-01174-3

**Published:** 2021-10-14

**Authors:** Keshu Zhou, Dehui Zou, Jianfeng Zhou, Jianda Hu, Haiyan Yang, Huilai Zhang, Jie Ji, Wei Xu, Jie Jin, Fangfang Lv, Ru Feng, Sujun Gao, Daobin Zhou, Constantine S. Tam, David Simpson, Michael Wang, Tycel J. Phillips, Stephen Opat, Zhiyue Huang, Huafei Lu, Yuqin Song, Yongping Song

**Affiliations:** 1 Affiliated Cancer Hospital of Zhengzhou University, Henan Cancer Hospital, Zhengzhou, 450003 China; 2 Institute of Hematology and Blood Diseases Hospital, Chinese Academy of Medical Sciences and Peking Union Medical College, Tianjin, 300020 China; 3 Tongji Hospital, Tongji Medical College, Wuhan, 430032 China; 4 Fujian Medical University Union Hospital, Fuzhou, 35000 China; 5 Zhejiang Cancer Hospital, Hangzhou, 310000 China; 6 Tianjin Medical University Cancer Institute and Hospital, Tianjin, 300060 China; 7 West China Hospital of Sichuan University, Chengdu, 610041 China; 8 The First Affiliated Hospital of Nanjing Medical University, Jiangsu Province Hospital, Nanjing, 210029 China; 9 The First Affiliated Hospital, Zhejiang University College of Medicine, Hangzhou, 310003 China; 10 Fudan University Shanghai Cancer Center, Shanghai, 200032 China; 11 Nanfang Hospital of Southern Medical University, Guangzhou, 510515 China; 12 The First Hospital of Jilin University, Changchun, 130031 China; 13 Peking Union Medical College Hospital, Chinese Academy of Medical Sciences and Peking Union Medical College, Beijing, 100730 China; 14 Peter MacCallum Cancer Centre, St. Vincent’s Hospital, University of Melbourne, Melbourne, VIC 3065 Australia; 15 North Shore Hospital, Auckland, 2065 New Zealand; 16 The University of Texas MD Anderson Cancer Center, Houston, TX 78701 USA; 17 University of Michigan, Ann Arbor, MI 48109 USA; 18 Monash Health, Monash University, Clayton, VIC 3800 Australia; 19 BeiGene (Beijing) Co., Ltd, Beijing, 102206 China; 20 Department of Lymphoma, Peking University Cancer Hospital and Institute, Beijing, 100142 China

**Keywords:** Complete response rate, Mantle cell lymphoma, Progression-free survival, Second-line therapy, Zanubrutinib

## Abstract

Mantle cell lymphoma (MCL) is a mature B-cell neoplasm with a high initial response rate followed almost invariably by relapse. Here we report the pooled data from 2 studies, BGB-3111-AU-003 and BGB-3111-206, to explore the efficacy of zanubrutinib monotherapy in relapsed/refractory (R/R) MCL. A total of 112 patients were included. Median follow-up durations were 24.7 and 24.9 months for BGB-3111-AU-003 and BGB-3111-206, respectively. Overall response rate (ORR) and complete response (CR) rate were 84.8% and 62.5%, and median duration of response, progression-free survival (PFS) and overall survival (OS) were 24.9, 25.8 and 38.2 months, respectively. After weighting, the PFS (median: NE vs. 21.1 months, *P* = 0.235) and OS (median: NE vs. 38.2 months, *P* = 0.057) were similar but numerically better in the second-line than later-line group. Zanubrutinib was well-tolerated with treatment discontinuation and dose reduction for adverse events in 12.5% and 2.7% of patients, respectively. Hypertension, major hemorrhage and atrial fibrillation/flutter rates were 11.6%, 5.4% and 1.8%, respectively. Zanubrutinib is efficacious in R/R MCL, with a favorable safety profile.

**Supplementary Information:**

The online version contains supplementary material available at 10.1186/s13045-021-01174-3.

To the editor,

MCL is a rare, B-cell non-Hodgkin lymphoma with highly heterogeneous clinical presentation and aggressiveness [1–3]. Before the use of Bruton’s tyrosine kinase (BTK) inhibitors, therapeutic options for patients with R/R MCL were limited, and their outcomes were generally poor [4–6]. Zanubrutinib is a next-generation, highly specific and potent BTK inhibitor [3, 7].Based on two phase I/II studies (BGB-3111-206 and BGB-3111-AU-003) [8, 9], Zanubrutinib was approved in 2019 by the US Food & Drug Administration for the treatment of adult patients with MCL who have received at least one prior therapy.

For this analysis, the patient-level data from BGB-3111-206 and BGB-3111-AU-003 were pooled to further characterize the efficacy profile of zanubrutinib monotherapy in R/R MCL.

A total of 112 patients were included, with 33 from BGB-3111-AU-003 and 79 from BGB-3111-206. The median duration of follow-up in BGB-3111-AU-003 and BGB-3111-206 was 24.7 and 24.9 months, respectively. Across the overall population, the median duration of follow-up was 24.9 months, and the duration of treatment was 20.4 months. Most of the patients had Stage III or IV disease (91%) and low to intermediate MCL International Prognostic Index (MIPI) risk scores (79%). There were 8% with bulky disease and 13% with blastoid variant (Table 1).

**Table 1:** Baseline covariates in two trials

	All (*n* = 112)	BGB-3111-AU-003 (*n* = 33)	BGB-3111-206 (*n* = 79)
Age			
Mean (SD)	61.55 (9.97)	69.12 (9.93)	58.39 (8.16)
Median	62	70	60
Sex, male	86 (77%)	25 (76%)	61 (77%)
BMI, mean (SD)	24.94 (4.18)	27.88 (4.82)	23.72 (3.19)
ECOG PS, > 1	6 (5%)	3 (9%)	3 (4%)
Disease stage			
I	3 (3%)	2 (6%)	1 (1%)
II	7 (6%)	0	7 (9%)
III	14 (13%)	1 (3%)	13 (16%)
IV	88 (79%)	30 (91%)	58 (73%)
Number of prior lines of therapy, median	2	1	2
Blastoid variant	14 (13%)	2 (6%)	12 (15%)
MIPI			
High risk	24 (21%)	15 (45%)	9 (11%)
Intermediate risk	33 (29%)	10 (30%)	23 (29%)
Low risk	55 (49%)	8 (24%)	47 (59%)
Bulky	9 (8%)	3 (9%)	6 (8%)
Extra-nodal	67 (60%)	9 (27%)	58 (73%)
Bone marrow involvement	58 (52%)	21 (64%)	37 (47%)

*BMI* body mass index, *ECOG PS* Eastern Cooperative Oncology Group performance status, *Bulky* longest transverse diameter of a lesion > 10 cm, *SD* standard deviation

Before weighting, there were 41 patients in the second-line group and 71 patients in the later-line group. The second-line group had higher age, body mass index (BMI) and a higher percentage of patients with high MIPI risk scores, and lower percentages of patients with extra nodal disease and blastoid subtype compared with the later-line group. After weighting, all baseline covariates were balanced between the second- and later-line groups (Additional file 1: Table S1).The effective sample sizes were 27 in the second-line group and 59 in the later-line group, with median treatment durations of 22 and 18.8 months, respectively.

Prior treatment regimens included cyclophosphamide/vincristine/doxorubicin/dexamethasone (hyper-CVAD) or hyper-CVAD-like regimens (9% and 19%), lenalidomide (0 and 14%), bortezomib (1% and 10%) and autologous stem cell transplantation (2% and 10%) in second- and later-line therapy groups, respectively. The percentage of patients who received prior bendamustine was low in both groups (4% in second-line and 5% in later-line; Additional file 1: Table S2).

In BGB-3111-AU-003, the ORR was 84.9%, and the CR rate was 24.2%; the median PFS was 16 months, and the median OS was 25.8 months. In BGB-3111-206, the ORR was 84.8%, and the CR rate was 78.5%; the median PFS was 25.8 months, and the median OS was not reached (Additional file 1: Figure S1). The difference in CR rates between the two trials might be due to the different imaging strategies (Additional file 1) and poorer patients’ condition in BGB-3111-AU-003 (Table 1). In the pooled population, the ORR and the CR rate were 84.8% (95% CI: 76.8–90.9%) and 62.5% (95% CI: 52.8–71.5%); the median duration of response(DOR), PFS and OS were 24.9 (95% CI: 19.5-not estimable [NE]), 25.8 (95% CI: 16.8-NE) and 38.2 (95% CI: 29.3-NE) months, respectively (Additional file 1: Figure S2).

After weighting, the ORR (89.4 vs. 85.5%, adjusted OR = 1.5; *P* = 0.538), DOR (median: NE vs. 23.1 months, adjusted HR = 0.743; *P* = 0.436), PFS (median: NE vs. 21.1 months, adjusted HR = 0.679; *P* = 0.235) and OS (median: NE vs. 38.2 months, adjusted HR = 0.449; *P* = 0.057)were similar but numerically better in the second-line than later-line group (Additional file 1: Figure S3).

In the original population, the rate of treatment discontinuation due to disease progression was 40.2% and due to AEs was 12.5%. Most patients (96.4%) experienced at least one AE, and 50.9% experienced at least one grade ≥ 3 AE. Serious AEs (SAEs) occurred in 33.9% of patients and AE leading to death occurred in 7.1% (congestive heart failure, n = 1; general disorders, n = 2; pneumonia, n = 2; road traffic accident, n = 1; hemorrhagic stroke, n = 1; ischemic stroke, n = 1). The most focused AE of special interest (AESI) were hypertension (11.6%), major hemorrhage (5.4%) and atrial fibrillation/flutter (1.8%). The incidence of grade ≥ 3 atrial fibrillation was 0.89% (Table 2). Detailed information of AEs was presented in Additional file 1: Table S3.

**Table 2:** Extent of exposure and adverse events before and after weighting

	Before weighting			After weighting		
	Second-line therapy (*n* = 41)	Later-line therapy (*n* = 71)	All (*n* = 112)	Second-line therapy (ESS = 27)	Later-line therapy (ESS = 59)	All (ESS = 86)
Extent of exposure						
Median duration of treatment (months)	20.53	20.27	20.4	22.0	18.8	19.9
Dose reduction due to AE, %	2.4	2.8	2.7	1.8	2.4	2.2
Dose interruption due to AE, %	4.9	14.1	10.7	3.7	11.2	8.5
Dose modification due to AE, %	7.3	14.1	11.6	5.5	11.2	9.2
Treatment discontinuation, %	51.2	56.3	54.5	46.9	58.3	54.3
Due to AE, %	17.1	9.9	12.5	10.6	11.0	10.9
Due to PD, %	34.1	43.7	40.2	36.4	42.9	40.6
Due to withdrawal, %	0.0	1.4	0.9	0.0	3.3	2.1
Due to investigators, %	0.0	1.4	0.9	0.0	1.1	0.7
Adverse events						
At least one AE, %	95.1	97.2	96.4	95.4	98.2	97.2
At least one grade ≥ 3 AE, %	51.2	50.7	50.9	47.3	48.1	47.8
At least one AE leading to death, %	4.9	8.5	7.1	3.1	7.9	6.2
At least one SAE, %	41.5	29.6	33.9	38.3	28.1	31.7
At least one AESI, %	78.1	91.6	86.6	82.5	91.3	88.2
Hypertension ^a^, %	12.2	11.3	11.6	12.2	12.7	12.5
Major hemorrhage ^b^, %	2.4	7.0	5.4	1.0	6.1	4.3
Atrial fibrillation/flutter	2.4	1.4	1.8	1.0	2.5	2.0
Grade ≥ 3 atrial fibrillation/flutter	0	1.4	0.9	0	2.5	1.6

*AE* adverse events, *AESI* adverse events of special interest, *PD* progressive diseases, *SAE* serious AE, *ESS* effective sample size

^a^Includes preferred terms hypertension and blood pressure increased

^b^Includes preferred term renal haematoma

In conclusion, zanubrutinib is an effective and well-tolerated therapeutic option for R/R MCL. Early treatment with zanubrutinib tends to have better survival profiles.

## Supplementary Information

**Additional file 1**.** Supplementary methods**.** Table S1**. Baseline covariates before and after weighting.** Table S2**. Prior medication uses after weighting.** Table S3**. Treatment emergent AEs (any grade, grade 3 or higher).** Figure S1**. Outcomes of patients with R/R MCL treated with zanubrutinib in the BGB-3111-AU-003 and BGB-3111-206 trial. (A) DOR in the BGB-3111-AU-003. (B) PFSin the BGB-3111-AU-003. (C) OS in the BGB-3111-AU-003. (D) DOR in the BGB-3111-206. (E) PFS in the BGB-3111-206. (F) OS in the BGB-3111-206.** Figure S2**. Outcomes of patients with R/R MCL treated with zanubrutinib. (A) DOR. (B) PFS. (C) OS.** Figure S3**. Outcomes of patients with R/R MCL treated with zanubrutinibas second- versus later-line therapy. (A) DOR before weighting. (B) PFS before weighting. (C) OS before weighting. (D) DOR after weighting. (E) PFS after weighting. (F) OS after weighting.

## Data Availability

The datasets used and/or analyzed during the current study are available from the corresponding author on reasonable request.

## Abbreviations

AEs: Adverse events
AESI: AE of special interest
BMI: Body mass index
BTK: Bruton’s tyrosine kinase
CR: Complete response
DOR: Duration of response
ECOG: Eastern Cooperative Oncology Group
EGFR: Epidermal growth factor receptor
FDA: Food & Drug Administration
IPSW: Inverse propensity score weighting
ITK: Interleukin-inducible tyrosine kinase
JAK3: Janus kinase 3
MCL: Mantle cell lymphoma
MIPI: MCL International Prognostic Index
NE: Not estimable
ORR: Overall response rate
OS: Overall survival
PFS: Progression-free survival
R/R: Relapsed/refractory
SAEs: Serious adverse events
