# Supplementary material for: Zanubrutinib monotherapy in relapsed/refractory mantle cell lymphoma: a pooled analysis of two clinical trials
Source: J Hematol Oncol. 2021 Oct 14;14:167. doi: 10.1186/s13045-021-01174-3 (PMC8518153; doi:10.1186/s13045-021-01174-3)
Supplement: Supplementary file 1 — Additional file 1. Supplementary methods. Table S1. Baseline covariates before and after weighting. Table S2. Prior medication uses after weighting. Table S3. Treatment emergent AEs (any grade, grade 3 or higher). Figure S1. Outcomes of patients with R/R MCL treated with zanubrutinib in the BGB-3111-AU-003 and BGB-3111-206 trial. (A) DOR in the BGB-3111-AU-003. (B) PFSin the BGB-3111-AU-003. (C) OS in the BGB-3111-AU-003. (D) DOR in the BGB-3111-206. (E) PFS in the BGB-3111-206. (F) OS in the BGB-3111-206. Figure S2. Outcomes of patients with R/R MCL treated with zanubrutinib. (A) DOR. (B) PFS. (C) OS. Figure S3. Outcomes of patients with R/R MCL treated with zanubrutinibas second- versus later-line therapy. (A) DOR before weighting. (B) PFS before weighting. (C) OS before weighting. (D) DOR after weighting. (E) PFS after weighting. (F) OS after weighting. [file 13045_2021_1174_MOESM1_ESM.docx]

**Additional file 1** Supplemental Material.

**Supplementary Methods**

***Study designs and patients***

This analysis included pooled data for patients with r/r MCL treated with zanubrutinib monotherapy in two Phase 2 studies: BGB-3111-AU-003 (NCT02343120) and BGB-3111-206 (NCT03206970). BGB-3111-AU-003 was a study of zanubrutinib monotherapy in patients with B-cell malignancies from Australia, Italy, South Korea, New Zealand, the United Kingdom and the United States. It was comprised of 2 parts: dose escalation and cohort expansion. The dose escalation part included dose levels from 40 mg per day to a maximum of 320 mg per day. Patients in the dose expansion cohorts then received 320 mg once daily (QD) or 160 mg twice daily (BID). In BGB-3111-206, Chinese R/R MCL patients received zanubrutinib at 160 mg BID. Previous analysis showed that similar plasma exposure and BTK inhibition were achieved between the two groups of 160 mg BID and 320 mg QD, and that differences in trough concentration and maximum plasma concentration are unlikely to have a meaningful impact on efficacy and safety endpoints [1].

All R/R MCL patients with no missing baseline covariate (age, sex, BMI, ECOG performance status, disease stage, blastoid variant, MIPI, bulky disease, extra-nodal disease, bone marrow involvement and the number of prior lines of therapy) were pooled; one patient in BGB-3111-206 was excluded at the investigator’s discretion. Patients were divided into two groups based on the zanubrutinib treatment line: second- and later-line. Bulky disease was defined as the longest transverse diameter of a lesion (LDi) >10 cm.

***Assessment***

Efficacy endpoints in both studies included CR rate, ORR, DOR, PFS, and OS. Response to treatment was assessed according to the Lugano classification [2]. Positron emission tomography-computed tomography (PET-CT) imaging was required of all patients with FDG-avid disease both at baseline and at all follow-up response assessments in Study BGB-3111-206. In BGB‑3111‑AU‑003, however, PET-CT was required only for confirmation of suspected CR. The higher CR rate in BGB-3111-206 compared to BGB‑3111‑AU‑003 is likely a result of the different imaging approaches that were used to assess response in the two studies (only four patients had on-study PET-CT in BGB-3111-AU-003). The difference in response assessment methods between the two trials may have impacted the pooled CR rate, and the CR rate was not compared between the second- and later-line groups.

AEs were graded based on NCI-CTCAE version 4.03. AESI included hemorrhage (major hemorrhage defined as intracranial hemorrhage, severe hemorrhage, grade ≥ 3 bleeding from other tissues or organs), atrial fibrillation and flutter, hypertension, second primary malignancies, tumor lysis syndrome, infection, and cytopenia.

***Statistical analysis***

Efficacy endpoints (ORR, CR rate, DOR, PFS and OS) and safety parameters (incidence and severity of AEs) were examined in the pooled sample, and in the second- and later-line groups (after balancing baseline covariates).

Inverse propensity score weighting (IPSW) was used to balance baseline covariates (age, sex, BMI, ECOG performance status, disease stage, blastoid variant, MIPI, bulky disease, extra-nodal disease and bone marrow involvement) between the second- and later-line groups [3]. Baseline covariates were used to create a propensity score model, along with the prior medication. The balance criteria for continuous variables were: 1) a standardized mean difference not exceeding 0.1; 2) a ratio of variances between 0.67 and 1.5. The balance criterion for binary variables was an absolute mean difference not exceeding 0.1 [4, 5]. The IPSW process was designed to preserve the original prevalence of prior medication use after weighting.

In addition, descriptive statistics for efficacy and safety parameters, as well as exploratory analyses, were conducted using Kaplan-Meier estimates for the efficacy endpoints DOR, PFS and OS. The difference in ORR between groups was investigated by logistic regression. The Cox proportional hazards model was used to evaluate the difference in time-to-event endpoints between groups. The difference was adjusted by the study as BGB-3111-AU-003 and BGB-3111-206 varied in race demographics (Asian vs. non-Asian cases) and response assessment methods (CT versus PET). All analyses were performed using R version 3.6.1 (Weight, It and Survey packages). This is a post-hoc analysis. *P*-values were reported for descriptive purpose.

**Table S1.** Baseline covariates before and after weighting

|  | Before weighting | | | After weighting | | |
| --- | --- | --- | --- | --- | --- | --- |
|  | Second-line therapy  (n=41) | Later-line therapy  (n=71) | Mean. diff.,  (var. ratio) | Second-line therapy  (ESS=27) | Later-line therapy  (ESS=59) | Mean. diff.,  (var. ratio) |
| Age, mean (SD) | 63.95 (11.45) | 60.17 (8.80) | 0.37 (1.69) | 61.20(9.67) | 61.23(9.69) | 0.00(1.00) |
| Sex, male | 33 (80%) | 53 (75%) | 0.06 | 78% | 77% | 0.02 |
| BMI, mean (SD) | 25.69 (4.05) | 24.51 (4.22) | 0.28 (0.92) | 24.65(3.69) | 24.73(4.26) | -0.02(0.75) |
| ECOG PS, >1 | 2(5%) | 4(6%) | -0.01 | 3% | 5% | -0.01 |
| Disease stage |  |  |  |  |  |  |
| I | 2 (5%) | 1 (1%) | 0.03 | 4% | 5% | -0.01 |
| II | 2 (5%) | 5 (7%) | -0.02 | 5% | 6% | -0.02 |
| III | 4 (10%) | 10 (14%) | -0.04 | 17% | 14% | 0.03 |
| IV | 33 (80%) | 55 (77%) | 0.03 | 75% | 76% | -0.01 |
| Blastoid variant | 1 (2%) | 13 (18%) | -0.16 | 2% | 12% | -0.11 |
| MIPI |  |  |  |  |  |  |
| High risk | 12(29%) | 12(17%) | 0.12 | 18% | 20% | -0.02 |
| Intermediate risk | 11(27%) | 22(31%) | -0.04 | 31% | 31% | 0.00 |
| Low risk | 18(44%) | 37(52%) | -0.08 | 51% | 49% | 0.02 |
| Bulky | 3 (7%) | 6 (8%) | -0.01 | 6% | 8% | -0.02 |
| Extra-nodal | 21 (51%) | 46 (65%) | -0.14 | 65% | 62% | 0.03 |
| Bone marrow involvement | 21 (51%) | 37 (52%) | -0.01 | 52% | 52% | 0.01 |

BMI, body mass index; ECOG PS, Eastern Cooperative Oncology Group performance status; LDi, longest transverse diameter of a lesion; SD, standard deviation; ESS: effective sample size.

**Table S2.** Prior medication uses after weighting

| Prior Medication Use | Second-line therapy | Later-line therapy |
| --- | --- | --- |
| (R) CHOP / (R) CHOEP / (R) CHOP-like | 75% | 88% |
| Rituximab or Rituximab Containing | 75% | 79% |
| Hyper CVAD or Hyper-CVAD-like | 9% | 19% |
| Lenalidomide | 0 | 14% |
| Bortezomib | 1% | 10% |
| Autologous stem cell transplantation | 2% | 10% |
| Bendamustine | 4% | 5% |

Hyper CVAD, cyclophosphamide, vincristine, doxorubicin and dexamethasone alternating with methotrexate and cytarabine; (R) CHOP, rituximab, cyclophosphamide, doxorubicin, vincristine and prednisone; (R) CHOEP, rituximab, cyclophosphamide, doxorubicin, vincristine, etoposide and prednisone.

**Table S3.** Treatment emergent adverse events (any grade, grade 3 or higher)

| Preferred Term | Any grade, n (%) | Grade >3, n (%) |
| --- | --- | --- |
| Alanine aminotransferase increased | 12 (10.7) | 1 (0.9) |
| Anemia | 19 (17.0) | 9 (8.0) |
| Constipation | 15 (13.4) | 1 (0.9) |
| Contusion | 13 (11.6) | 0 |
| Cough | 15 (13.4) | 0 |
| Diarrhea | 26 (23.2) | 1 (0.9) |
| Hypertension | 12 (10.7) | 3 (2.7) |
| Hypokalemia | 15 (13.4) | 1 (0.9) |
| Lung infection | 9 (8.0) | 7 (6.3) |
| Neutrophil count decreased | 36 (32.1) | 14 (12.5) |
| Platelet count decreased | 27 (24.1) | 8 (7.1) |
| Pneumonia | 6 (5.4) | 4 (3.6) |
| Rash | 33 (29.5) | 0 |
| Upper respiratory tract infection | 39 (34.8) | 0 |
| Urinary tract infection | 13 (11.6) | 1 (0.9) |
| White blood cell count decreased | 25 (22.3) | 6 (5.4) |

Any grade events that occurred in at least 10% of patients and grade ≥3 events occurred in at least 3% of patients.

**Figure S1**

**
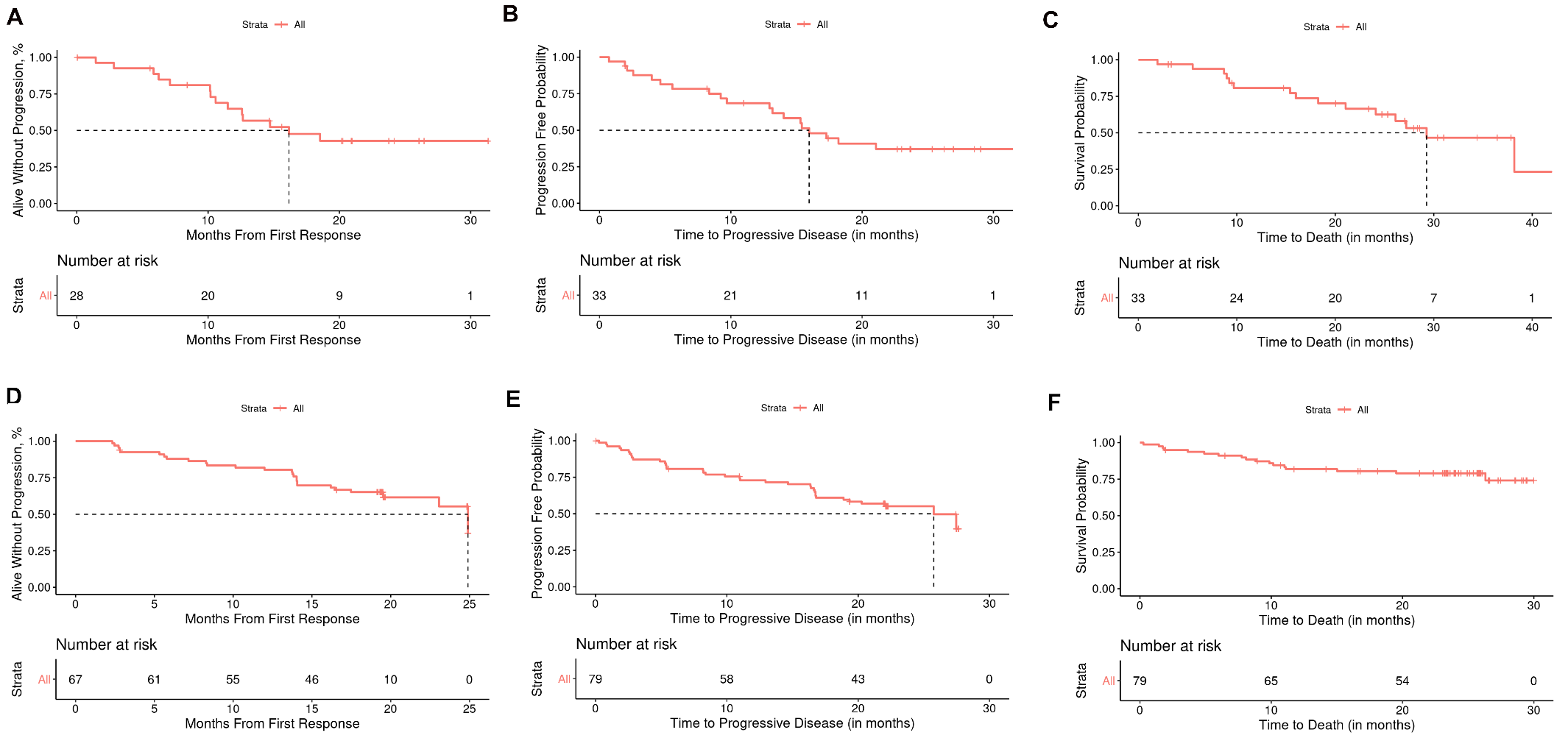
**

**Figure S2**

**
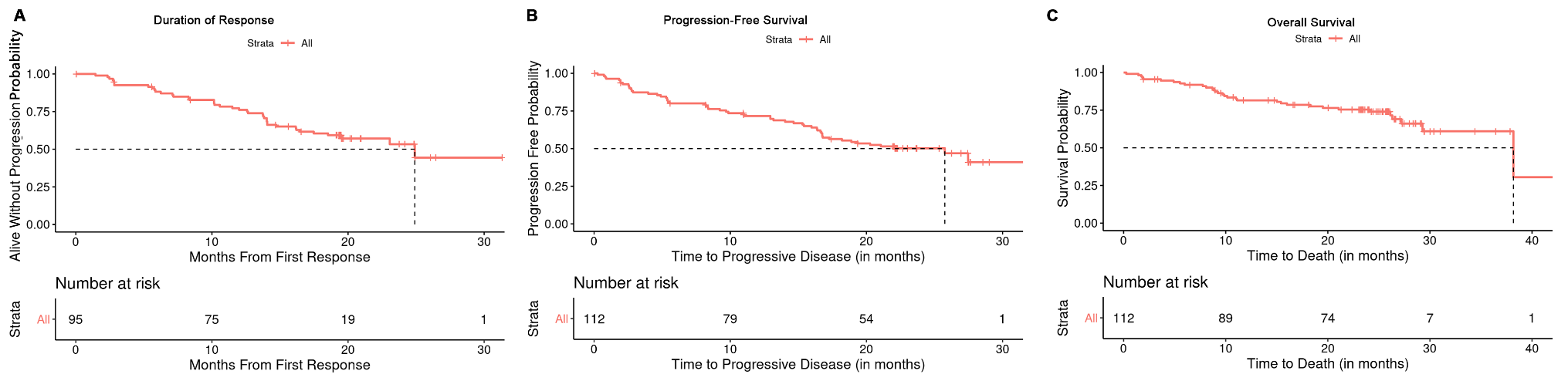
**

**Figure S3**

**
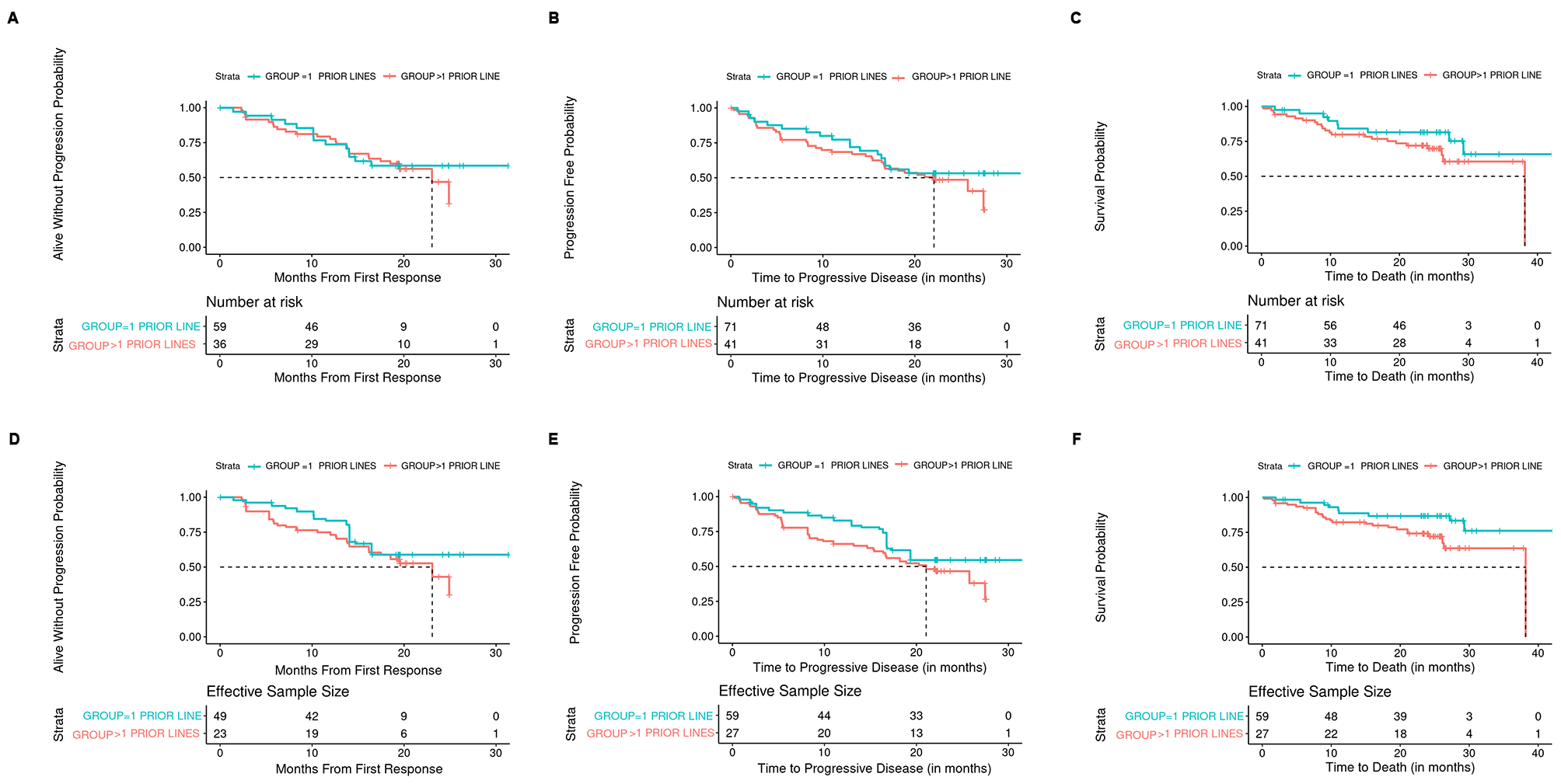
**

**References**

1. Ou YC, Tang Z, Novotny W, Cohen A, Wang K, Liu L, et al. Rationale for once-daily or twice-daily dosing of zanubrutinib in patients with mantle cell lymphoma. Leuk Lymphoma. 2021:1-13.

2. Cheson BD. Staging and response assessment in lymphomas: the new Lugano classification. Chin Clin Oncol. 2015;4:5.

3. Raad H, Cornelius V, Chan S, Williamson E, Cro S. An evaluation of inverse probability weighting using the propensity score for baseline covariate adjustment in smaller population randomised controlled trials with a continuous outcome. BMC Med Res Methodol. 2020;20:70.

4. Yang D, Dalton JE. A unified approach to measuring the effect size between two groups using SAS®. 2012.

5. Austin PC. Using the Standardized Difference to Compare the Prevalence of a Binary Variable Between Two Groups in Observational Research. Communications in Statistics - Simulation and Computation. 2009;38:1228-34.
